# Discordance Between Stimulated and Spontaneous Growth Hormone Levels in Short Children Is Dependent on Cut-Off Level and Partly Explained by Refractoriness

**DOI:** 10.3389/fendo.2020.584906

**Published:** 2020-11-17

**Authors:** Otto Lennartsson, Ola Nilsson, Maria Lodefalk

**Affiliations:** ^1^Department of Pediatrics, Örebro University Hospital, Örebro, Sweden; ^2^Department of Pediatrics, Faculty of Medicine and Health, Örebro University, Örebro, Sweden; ^3^Division of Pediatric Endocrinology and Center for Molecular Medicine, Karolinska Institutet and University Hospital, Stockholm, Sweden

**Keywords:** arginine-insulin tolerance test, children, growth hormone, GH testing, GH deficiency, refractoriness, rhGH therapy, short stature

## Abstract

**Background:**

A growth hormone (GH) stimulation test is the recommended method for evaluating GH levels in children with possible GH deficiency (GHD). However, serial measurements of nocturnal spontaneous GH secretion are also performed. Divergent results from these tests have been reported, but with variable frequencies.

**Objectives:**

To investigate whether performing one or two GH tests is associated with the probability to diagnose a child with GHD; the frequency of divergent results in the arginine-insulin tolerance test (AITT) and the nocturnal spontaneous test using different cut-off levels, and whether refractoriness may explain some of the discordance.

**Methods:**

In a population-based setting, the medical records of all short children evaluated for possible GHD during January 1993–February 2017 were reviewed. Twenty-one patients had been evaluated with one GH test only and 102 children had been evaluated with a spontaneous nocturnal GH test followed immediately by a complete AITT. Divergent results were defined as having a pathological response on only one of the tests when using 3, 5, 7, and 10 µg/L as cut-offs for peak GH on both tests, 1.1 and 3.3 µg/L for mean nocturnal values and receiver operating characteristic curves-derived cut-offs for nocturnal values.

**Results:**

Children evaluated with one test only were more often diagnosed with GHD compared with children evaluated with both tests (48 vs. 19%, p = 0.019). Divergent results were found in 6–42% of the patients, with higher frequencies seen when higher cut-offs were applied. A higher proportion of patients with stimulated peak values ≤ 7 and ≤ 5 µg/L had a spontaneous peak within 2 h before the start of the AITT compared with patients with higher stimulated peak values (68 vs. 45%, p = 0.026, and 77 vs. 48%, p = 0.033, respectively).

**Conclusions:**

Divergent results between AITT and nocturnal spontaneous secretion are common in short children, dependent on the cut-offs applied and partly due to refractoriness. Performing both tests decreases the risk of over diagnosing GHD in short children.

## Introduction

Growth hormone (GH) status can be assessed by serial sampling (every 20–30 min during the night or during 24 h) or after GH stimulation. Both methods—the spontaneous test and the stimulation test—have well-known shortcomings (1), including poor reproducibility (2). A GH stimulation test, using at least two different stimuli, is the recommended mode of testing for GH deficiency (GHD) in children in both current and previous international guidelines (3, 4). However, it is likely that combining the spontaneous test with a stimulation test would increase the magnitude of the highest GH peak detected and therefore decrease the frequency of GHD diagnoses. However, this hypothesis is incompletely studied.

The correlation between different GH stimulation tests (2, 5, 6), as well as between stimulated and spontaneous GH levels, is poor to moderate. Earlier studies have shown that divergent results, i.e. only one of the tests shows pathological values, occur in a relatively large fraction of the patients. However, the reported proportion of children with divergent results between spontaneous and stimulated GH peaks varies substantially in different studies: in nine out of 62 children (14%) (7), in six of 37 children (16%) (8), and in 62 of 116 children (53%) (9). Yet another study found that five of 30 short children (17%) had normal spontaneous 24 h GH secretion but abnormal stimulation test results (10). Furthermore, poor correlations between stimulated GH peak values and 24 h mean GH values were found in patients (aged 3.5–20.6 years) diagnosed with GHD or GH neurosecretory dysfunction and in short control children, in total 60 patients (11). However, the spontaneous test was performed several months after the stimulation test in that study, which might have influenced the results as both nutritional and pubertal status might have changed during that time interval and these factors have a profound influence on GH secretion (12, 13). A modest correlation between 24 h integrated GH concentrations and stimulated GH peak concentrations was also found in 90 short patients (aged 5–20 years) and 33 of 71 short patients (46%) with normal stimulated GH peaks had low 24 h GH values (14). In addition, five of 23 (22%) healthy female adolescents did not respond normally to an exercise test even though they were not short and they had normal spontaneous nocturnal GH secretion (15). However, the study populations in these earlier reports have often been limited in size, not restricted to a well-described geographical area, and different studies have employed different cut-offs making comparison between them hard. To the best of our knowledge, no earlier study has investigated the prevalence of divergent results between a spontaneous and a stimulation test when applying several, different cut-off values to the same study population.

It is probable that the chosen cut-off value influences the prevalence of divergent results between the spontaneous and the stimulation test as it is harder to pass a GH test using higher cut-offs. The cut-off discriminating a pathological GH response to stimulation from a normal response is debated and has varied over time from 3.5 to 10 µg/L (16). Nowadays, the proposed cut-off value is close to 7 µg/L, but a valid and exact cut-off value does not exist (16, 17). Furthermore, different stimuli elicit variable GH responses (18) and older GH assays yielded higher values than newer assays (17). The cut-off value for the spontaneous nocturnal test is neither well defined, but some clinicians use the same cut-off value as for stimulated GH peaks (19). However, according to previous reports, 3 µg/L in GH peak concentration and 1.1 or 3.3 µg/L in mean GH concentration may be suitable cut-offs in the spontaneous nocturnal test (8, 9, 20, 21).

Another possible reason for divergent results between spontaneous and stimulated GH secretion may be refractoriness during the stimulation test. A refractory interval was described for the first time in 1976 as the absence of a GH response to repeated oral levodopa stimulation in 16 healthy adults (22). The GH response to a repeated dose returned to the initial level only 6 h after the first dose. However, this time interval was reduced to 5 h with doubled levodopa doses. Furthermore, above-lactate threshold exercise on a cycle ergometer during 10 min and repeated twice, 1 and 2 h after the first exercise bout, resulted in clearly attenuated GH responses to the second and third exercise bouts in nine healthy adults (23). This finding was further investigated in 23 healthy adolescent females (15–17 years of age) immediately after a nocturnal spontaneous GH test (15). Five of them did not respond to an exercise test and all non-responders had had a spontaneous GH peak within 60 min before the stimulation test whereas none of the 18 participants responding to the exercise test had had a spontaneous GH peak within 80 min before the stimulation test. However, as far as we know, no study has yet described a GH refractory interval in short children aged < 15 years nor in any humans during other stimulation than levodopa or exercise, for example during the arginine-insulin tolerance test (AITT).

The aims of the present study were therefore to 1) investigate in a clinical setting whether diagnosing short children with GHD is associated with number of GH tests performed; 2) assess the frequency of divergent results in the spontaneous nocturnal and the stimulation tests using different cut-off values; 3) identify the best cut-offs for spontaneous nocturnal GH peak and mean values according to different stimulated cut-off values; and 4) investigate whether refractoriness is associated with the results of GH stimulation in short children.

## Materials and Methods

### Subjects

Eligible patients for the present study were children (0–18 years of age) who had been evaluated for possible GHD by measuring nocturnal spontaneous GH secretion, stimulated GH secretion or both types of secretion at the Department of Pediatrics at Örebro University Hospital, Örebro, Sweden, from the 1^st^ of January 1993 until the 28^th^ of February 2017. This department is the sole referral center for the evaluation of GHD in children living in Örebro County, which is situated in Middle Sweden and has a population of approximately 300,000. At this department, children with suspected GHD have routinely been investigated by measuring nocturnal spontaneous GH secretion followed by an AITT (24). However, at the discretion of the responsible pediatric endocrinologist, sometimes only one of the tests was performed, and sometimes only one stimulus was given in the stimulation test. Priming with sex steroids prior to GH testing in prepubertal children was not done routinely.

Despite the long time period, only two pediatric endocrinologists were involved in the assessments of all patients. All outpatient visits and inpatient stays at the hospital are routinely and prospectively registered with the patient’s ICD code and the code numbers for procedures performed. These hospital registries were searched for eligible patients for this study. The ICD-9 and 10 codes and procedure code numbers used for the search can be found in **Supplementary Material**(**Table S1**). To confirm that all eligible patients were identified, the Department of Clinical Chemistry registry was also searched for 0–18-year-old patients who had had at least one blood sample analyzed for a GH concentration during the study period.

**Figure 1:**
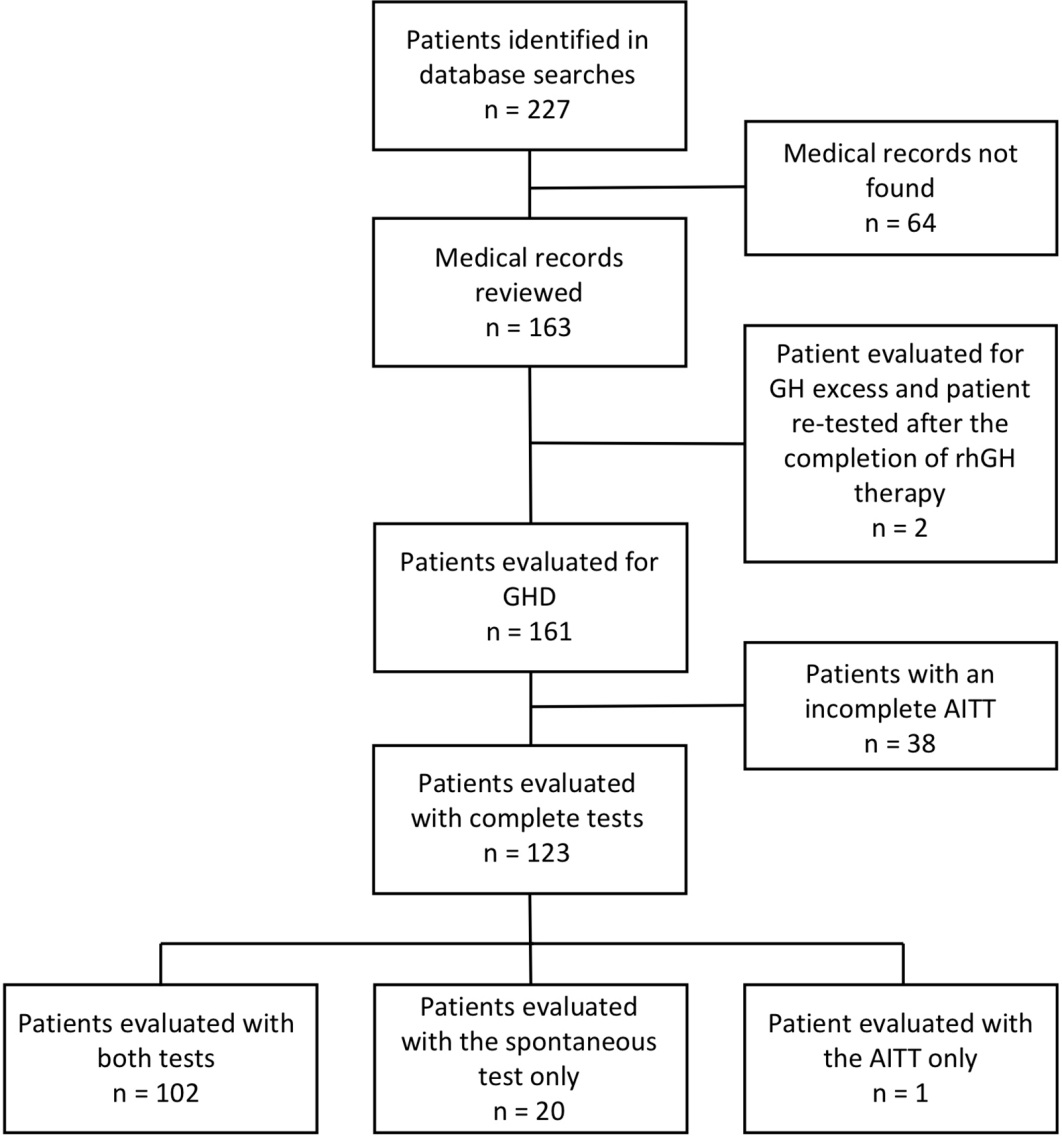
Flowchart of included and excluded children with short stature evaluated for possible GHD. AITT, arginine-insulin tolerance test. GH, growth hormone. GHD, growth hormone deficiency.

The search identified 227 individuals. The medical record from the Department of Pediatrics was available for 163 of them. After the review of these records, one patient was excluded since she had been evaluated for suspected GH excess and another patient was excluded as his GH investigation represented a retesting after a period of rhGH treatment to evaluate remaining GHD. Thirty-eight patients were excluded as they had an incomplete AITT (only one stimulus was given or lack of adequate hypoglycemia (defined as ≤ 2.7 mmol/L)). The rest of the patients were included in the study (n = 123). Twenty patients were evaluated with the nocturnal test only and one patient was evaluated with the AITT only (**Figure 1**). Data on clinical characteristics, investigations performed, diagnosis given after testing (GHD or not), and whether rhGH therapy was initiated were collected in a structured way from the medical records. The data capture form can be found in **Supplementary Material**. Seventy-six patients were boys (62%). Eleven patients (9 boys) were primed with sex steroids prior to GH testing. The total population of children (0–18 years of age) living in Örebro County, Sweden, was 62,635 in 2000 and 62,156 in 2015 (25). The study was approved by the Regional Board of Ethics, Uppsala, Sweden (registration number: 2017/358).

### Procedures and Definitions

For the nocturnal spontaneous test, serial sampling was performed every 30 min from 8 PM until 8 AM the next morning and at 8:30 AM, the AITT started. The stimulation test was performed as described previously (24). Briefly, a 30 min intravenous (IV) infusion of arginine (0.5 g/kg body weight) was followed by an IV injection of insulin (0.1 IU/kg body weight). Younger children were given a lower dose of insulin (0.05–0.08 IU/kg body weight). Blood samples were collected at 0, 15, 30, 45, 60, 75, 90, 105, 120, 135, and 150 min after the start of the arginine infusion. All patients fasted during both nocturnal sampling and the AITT.

For the calculations of mean nocturnal GH concentrations, all values from 8 PM through 8 AM were included in accordance with similar studies (9, 11). However, the GH concentration at 8:30 AM was also considered a spontaneous value since no stimulus had been given prior to that time. Target height was calculated as the mean parental height plus 6.5 cm for boys or minus 6.5 cm for girls. Standard deviation scores (SDS) for height, weight, and target height were calculated based on Swedish pediatric growth reference data (26).

A divergent result in the two tests (spontaneous nocturnal test vs. AITT) was defined as only one of them showed normal values. Further, a normal response was defined as having at least one GH value above the chosen cut-off for peak GH concentrations. For the nocturnal test, a normal response was also defined as having a mean value above the chosen cut-off level for mean nocturnal concentrations. The following values were applied in the present study as cut-offs for peak GH concentrations in both tests: 3, 5, 7 and 10 µg/L in accordance with the literature (16, 17, 19, 20). For mean nocturnal GH concentrations, 1.1 and 3.3 µg/L were also applied as cut-off values in this study, as suggested before (8, 9, 20, 21). In addition, receiver operating characteristic (ROC) curves were created to identify suitable nocturnal spontaneous cut-off levels in our population when using different stimulated cut-off levels as diagnostic discriminators. The patients were then divided into four groups based on their results on the GH tests. Group A: Patients with normal results on both tests. Group B: Patients with pathological results on both tests. Group C: Patients with a pathological result only on the nocturnal test, and finally, Group D: Patients with a pathological result only on the stimulation test. The patients were not reclassified as GHD or not according to any specified criteria in this study. Instead the diagnoses given in the clinical practice after GH testing were retrieved from the medical records and these diagnoses are reported in the study.

### Laboratory Analyses

Trained personnel at the Department of Clinical Chemistry, Örebro University Hospital, performed all GH concentration analyses. The assay methods and standard preparations changed during the study period, as shown in **Supplementary Material** (**Table S2**). Before the 2^nd^ of March 2009, the unit for GH concentrations was mIU/L, and thereafter, the unit was µg/L. GH values in mIU/L were transformed to µg/L in the present study by dividing the values by 3, as recommended by the Department of Clinical Chemistry. GH concentrations below the detection limit (< 0.05 µg/L) were set at 0.03 µg/L, and GH concentrations above the upper limit (> 40 µg/L) were set at 40.1 µg/L.

Insulin-like growth factor-1 (IGF-1) concentrations were analyzed at the Department of Laboratory Medicine at Uppsala University Hospital, Uppsala, Sweden, before March 2010 and during April 2013–January 2014 and at the Department of Clinical Chemistry, Örebro University Hospital all other time periods. The Immulite^®^ (Siemens) assay was used for the analyses of IGF-1 levels during the whole study period except for April 2013 – January 2014 when the Diasorin^®^ (Liaison) assay was used. The reason for the change in assay methods was an insufficient supply of the antibodies needed for the Immulite^®^ method. The IGF-1 concentrations were interpreted as low, adequate or high according to reference values valid at the time of measurement, taking the patient’s sex, age and pubertal stage into account. Approximate SDS values were calculated using the IGF-1 SDS calculator provided by LabCorp^®^, taking the same variables into account (27).

### Statistical Analysis

Data are shown as the mean ± SD, median (min–max) or absolute numbers (percent). The Shapiro-Wilks test was used for normality testing. ANOVA, the Kruskal-Wallis test, the Student’s t test and the Mann-Whitney U test were used when comparing continuous variables between groups, as appropriate. Proportions were compared using the chi-square test or Fisher’s exact test, as appropriate. Pearson’s correlation test was used when analyzing correlations between variables. A logistic regression analysis was performed for the adjustment of potential confounding factors on associations between variables. Binomial analysis was performed for the comparison of proportions with divergent results at different cut-off levels. ROC curves were created to determine the correlation between the spontaneous nocturnal test and the AITT using different cut-offs on the AITT as discriminators. Statistical analyses were performed in SPSS, version 25 (IBM Corporation^®^, Armonk, NY, USA). Statistical significance was set at p < 0.05 for two-sided tests.

## Results

### The Probability to be Diagnosed With GHD in Relation to Number of Tests Performed

Children evaluated with one test only were more likely to be diagnosed with GHD compared with children evaluated with both tests (48 vs. 19%, p = 0.019, **Table 1**). The association between being diagnosed with GHD or not and being evaluated by one or two tests was statistically significant both before and after adjustment for age, pubertal stage, priming and sex as analyzed in a regression model (unadjusted B coefficient = 1.399, p = 0.007; adjusted B coefficient = 1.436, p = 0.012).

**Table 1:** Clinical characteristics of short children evaluated for possible growth hormone deficiency (GHD) divided by number of GH tests performed together with data on whether GHD was diagnosed after testing.

	Both tests performed completely (n = 102)	Single test performed completely (n = 21)	P value
Males:Females (% males)	60:42 (58%)	16:5 (76%)	0.136
Age (years)	8.8 (2.5–15.4)	5.7 (1.1–16.0)	0.106
Decade for the testing			0.527*
1990s	4 (4%)	1 (5%)	
2000s	32 (31%)	8 (38%)	
2010s	66 (65%)	12 (57%)	
Target height SDS	-1.4 (-3.8 – 0.7)	-0.6 (-2.0 – 0.7)	0.004
Weight SDS	-2.5 (-5.0 – 0.3)	-2.6 (-5.0 – 0.8)	0.857
Height SDS	-3.1 (-4.5 – 0.0)	-3.0 (-4.0 – 0.8)	0.678
BMI SDS	-0.1 (-4.0 – 2.3)	-0.4 (-4.5 – 2.4)	0.488
Pubertal stage			0.039**
*Tanner stage 1*	75 (82%)	20 (100%)	
*Tanner stage 2*	10 (11%)	0	
*Tanner stage 3*	6 (7%)	0	
*Tanner stage 4*	1 (1%)	0	
*Tanner stage 5*	0	0	
Primed before GH testing	9 (9%)	2 (10%)	>0.999
Diagnosed with GHD after testing	19 (19%)	10 (48%)	0.019

The values are median (min–max) or absolute number (percent of patients within group (both or single test), except for decade of GH investigation where the percent indicates the proportion of patients within each decade). Information on pubertal stage at the time of GH testing was available for only 112 patients.

GH, growth hormone. GHD, growth hormone deficiency. SDS, standard deviation score.

*Due to too few patients tested in the 1990s, only the number of patients tested in 2000s was compared with the number of patients tested in 2010s in the statistical analysis.

**Pubertal stage was dichotomized (Tanner stage 1 vs. Tanner stages 2–5) in the statistical analysis.

### Divergent Results in Nocturnal Spontaneous GH Test and AITT

**Figure 2:**
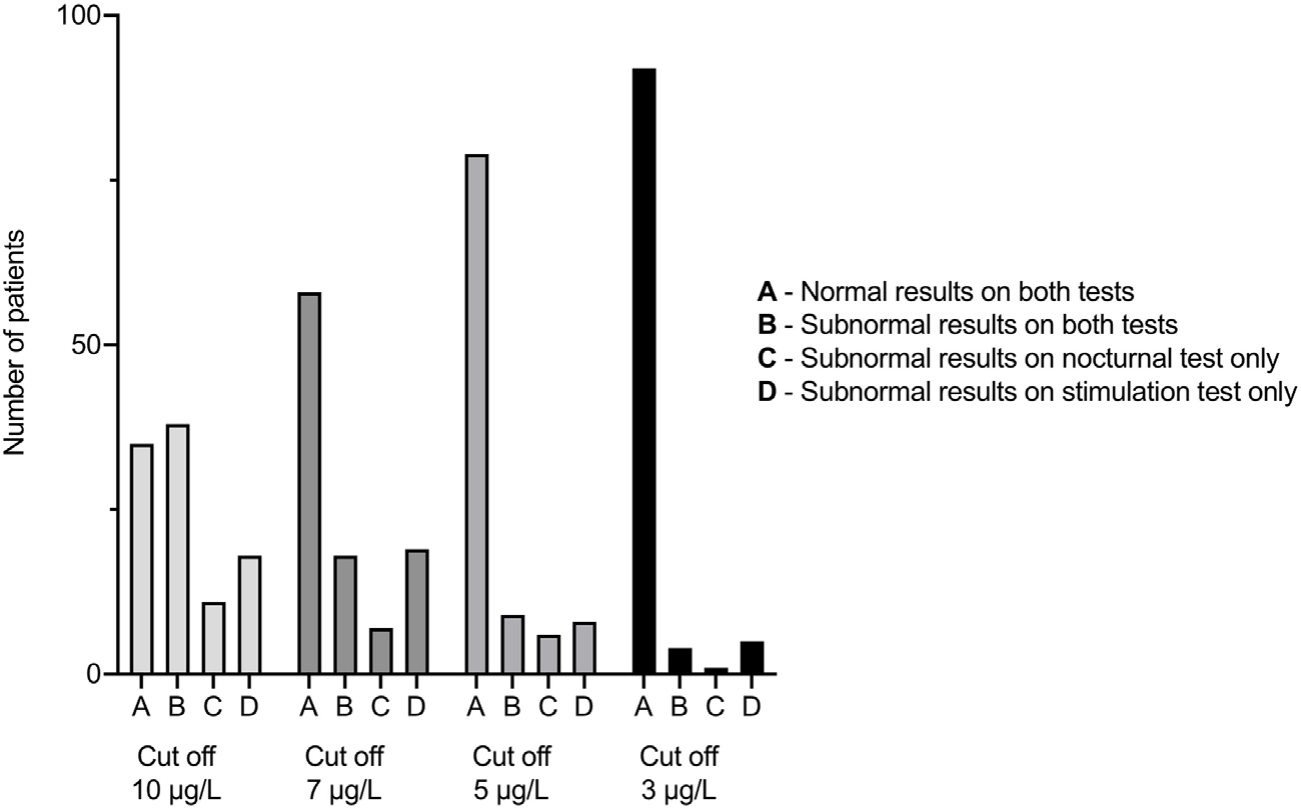
Number of children with short stature evaluated for possible GHD by both a nocturnal spontaneous GH test and an AITT in groups A–D, applying different cut-off levels for peak GH values. AITT, arginine-insulin tolerance test. GH, growth hormone. GHD, growth hormone deficiency.

The proportions of patients with divergent results on the two GH tests varied from 6–42% according to the cut-off level applied. The following proportions of patients had normal results on both tests (group A), pathological results on both tests (group B), pathological results on the nocturnal test only (group C), and pathological results on the stimulation test only (group D): 34, 37, 11, and 18%, respectively, when using 10 µg/L as the cut-off for peak GH levels on both tests; 57, 18, 7, and 19%, respectively, when using 7 µg/L as the cut-off for peak GH; 78, 9, 6, and 8%, respectively, when using 5 µg/L as the cut-off, and 90, 4, 1, and 5%, respectively, when using 3 µg/L as the cut-off for peak GH on both tests (**Figure 2**). The proportion of patients with divergent results when using 3 µg/L as the cut-off for peak GH levels in both tests differed statistically significant from that found when using 5 µg/L as the cut-off (p = 0.003). The same was true when comparing proportions of patients with divergent results using 5 µg/L as the cut-off with that found when using 7 µg/L as the cut-off (p = 0.002). The characteristics of the patients in groups A through D based on 7 µg/L as the cut-off for peak GH levels are shown in **Table 2** together with information on whether rhGH therapy was initiated or not.

**Table 2:** Clinical characteristics of short children evaluated with both a nocturnal spontaneous GH test and an AITT. The patients are divided into groups based on having concordant or divergent results in the tests when applying 7 µg/L as the arbitrary cut-off for peak GH values in both tests.

Group **Number of children (%)**	A58 **(57%)**	B18 **(18%)**	C7 **(7%)**	D19 **(19%)**	P value
Males:Females (% males)	28:30 (48%)	14:4 (78%)	4:3 (57%)	14:5 (74%)	0.212*
Age (years)	9.5 (2.5 – 15.4)	10.0 (4.4 – 13.7)	6.3 (3.9 – 10.5)	8.4 (3.2 – 15.0)	0.073
Target height SDS	−1.4 (−3.8 – 0.2)	−1.8 (−2.7 – −0.5)	−1.1 (−1.6 – 0.7)	−1.1 (−2.5 – 0.2)	0.119
Weight SDS	−2.7 (−5.0 – 0.2)	−2 (−3.3 – 0.3)	−2.4 (−5.0 – −1.2)	−2 (−3.0 – 0.0)	0.021
Height SDS	−3.2 (−4.5 – 0.0)	−3.2 (−4.0 – −2.0)	−3.0 (−4.0 – −1.9)	−3.0 (−4.0 – −2.4)	0.652
BMI SDS	−0.3 (−4.0 – 2.3)	−0.2 (−2.0 – 1.9)	−0.3 (−3.2 – 1.3)	0.4 (−1.2 – 2.1)	0.016
Height SDS minus target height SDS	−1.8 (−3.8 – 0.6)	−1.5 (−2.7 – 1.6)	−2.1 (−2.9 – −0.8)	−2.0 (−3.9 – −0.3)	0.201
IGF-1 below reference range	18 (31%)	6 (33%)	3 (43%)	6 (32%)	0.905**
Time point for the latest spontaneous peak	06:00 (0:00–08:30)	07:00 (04:30–08:30)	06:30 (05:30–07:30)	06:30 (04:00–07:30)	0.037
Time between the latest spontaneous peak and the first stimulated peak (min)	202.5 (60–540)	142.5 (90–285)	180 (120–240)	210 (90–330)	0.216
rhGH therapy initiated	14 (24%)	16 (89%)	5 (71%)	10 (53%)	<0.001***

The values are median (min–max) or absolute number (percent of valid patients within each group). Group A = Normal results on both tests. Group B = Pathological results on both tests. Group C = Pathological result on the nocturnal test only. Group D = Pathological result on the stimulation test only as defined in Method section.

AITT, arginine-insulin tolerance test. GH, growth hormone. IGF-1, insulin-like growth factor-1. SDS, standard deviation score.

*The chi-square test was used to compare patients in groups A and B with patients in groups C and D.

**The Fisher’s exact test was used to compare patients in group A with patients in groups B, C and D.

***The chi-square test was used to compare patients in group A with patients in groups B, C and D.

When 3.3 µg/L was applied as the cut-off for the mean concentration on the nocturnal test and 10 or 7 µg/L as the cut-off for stimulated peak GH, 26 (32%) and 34 (42%) patients showed divergent results on the two tests, respectively. Most of the patients with divergent results had low values on the nocturnal test only (Group C), irrespective of the chosen cut-off level for the stimulation test. Nine (11%) or seven (9%) patients had divergent results using 1.1 µg/L as the cut-off for the mean GH value on the nocturnal test and 5 µg/L or 3 µg/L as the cut-off for stimulated peak GH, respectively.

**Figure 3:**
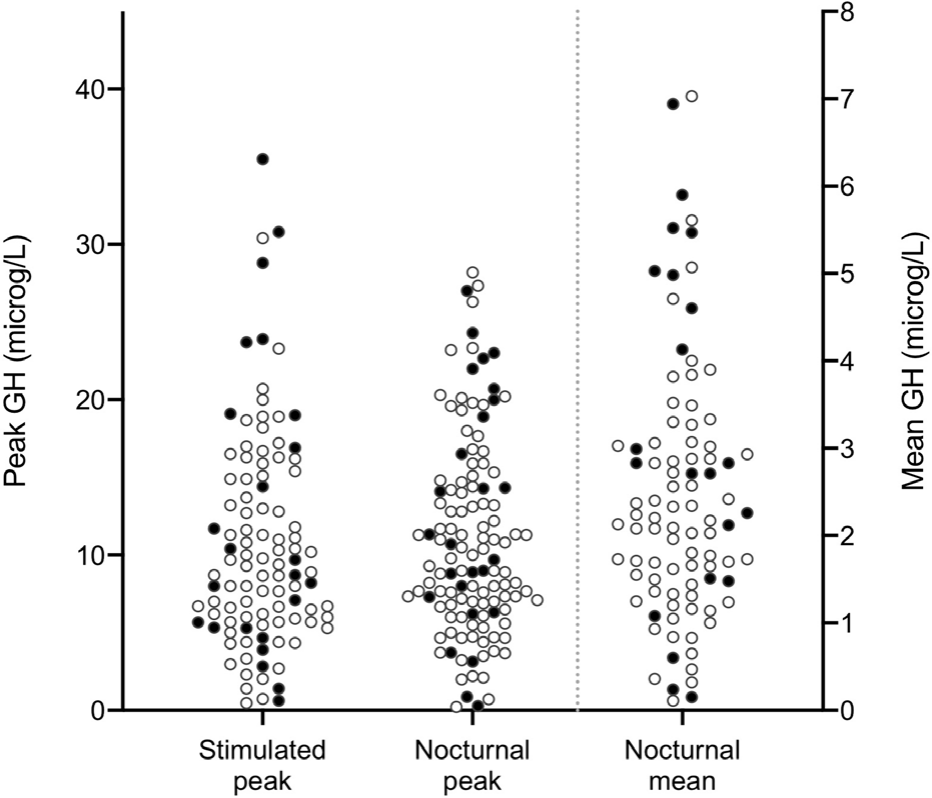
Scatter plots of maximum peak stimulated (AITT) and nocturnal spontaneous GH values and nocturnal mean values (µg/L) in 123 children with short stature evaluated for possible GHD. AITT, arginine-insulin tolerance test. GH, growth hormone. GHD, growth hormone deficiency. Open circles represent prepubertal patients and filled circles represent pubertal or primed patients.

In the whole study population, the nocturnal mean GH concentration was 2.2 µg/L (0.1–7.0 µg/L), and the maximum peak GH values during the nocturnal and the stimulation tests were 9.8 µg/L (0.2–28.2 µg/L) and 9.3 µg/L (0.5–35.5 µg/L), respectively (ns) (**Figure 3**). Stimulated GH peak values correlated with nocturnal peak values (r = 0.654, p < 0.001). Stimulated peak values also correlated with nocturnal mean values (r = 0.736, p < 0.001), and nocturnal peak GH values correlated with nocturnal mean values (r = 0.834, p < 0.001). To investigate whether an association existed between GH values and IGF-1 SDS, only prepubertal patients and patients without priming were included in the analysis as both GH and IGF-1 values increase with puberty. IGF-1 SDS was correlated with nocturnal peak GH values [r = 0.401, p = 0.001 (n = 66)] and nocturnal mean GH values [r = 0.374, p = 0.008 (n = 49)] in this subpopulation, but not significantly with stimulated peak GH values [r = 0.234, p = 0.083 (n = 56)].

### Calculated Cut-Off Values for the Nocturnal Spontaneous Test

In order to define cut-off values for nocturnal spontaneous GH secretion, ROC analyses with AITT as reference method were performed. Interestingly, all suggested cut-off values for peak GH concentrations in the nocturnal spontaneous test were higher than the corresponding cut-off peaks in the AITT (**Table 3**). However, using these calculated cut-offs for the nocturnal test together with the corresponding cut-offs for the AITT only marginally improved congruence of the tests as the fraction of divergent results remained high at 9–30% (**Table 3**).

**Table 3:** Results from ROC analyses of nocturnal peak and mean GH concentrations in short children when applying different cut-off levels for stimulated peak GH as diagnostic discriminators.

Cut-off level for stimulated peak GH (µg/L)	10	7	5	3
Best cut-off level for nocturnal peak GH level (µg/L)	10.5	7.8	7.2	4.7
AUC	0.775	0.760	0.862	0.925
Proportions with divergent results when applying the respective cut-off pairs	29.4%	26.5%	17.6%	8.8%
Best cut-off level for nocturnal mean GH level (µg/L)	2.1	1.9	1.4	1.2
AUC	0.775	0.799	0.876	0.927
Proportions with divergent results when applying the respective cut-off pairs	27.2%	22.8%	10.9%	8.7%

AUC, area under the curve. GH, growth hormone. ROC, receiver operating characteristic.

### Refractoriness

A higher proportion of the patients who had a stimulated GH peak ≤ 7 µg/L had a spontaneous GH peak within 2 h before the start of the AITT compared with the patients who had a stimulated GH peak > 7 µg/L (68 vs. 45%, p = 0.026). The same was true when applying 5 µg/L as the cut-off in the AITT (77 vs. 48%, p = 0.033). However, similar differences were not found when extending the time period prior to the stimulation test to 3 h.

The time point for the latest spontaneous GH peak differed statistically significant between patients in groups A through D when applying 7 µg/L as the cut-off for peak GH levels in both tests (p = 0.037, see **Table 2**). The average time point for the latest spontaneous GH peak was 30–60 min earlier in patients responding in both tests (Group A). However, the magnitude of the last spontaneous GH peak was higher in AITT responders compared with AITT non-responders when using 7 µg/L as the cut-off [4.6 µg/L (0.4–22.0) and 2.0 µg/L (0.15–12.3), respectively, p = 0.007].

## Discussion

In this population-based study, we found that a higher proportion of the children evaluated by one test only was diagnosed with GHD in the clinical setting compared with children evaluated by both a spontaneous and a stimulation test (48 vs. 19%). This association remained after adjusting for potential confounding factors indicating its robustness. The finding points at a risk for over diagnosing GHD if only one GH test is performed, even though two stimuli were given in the stimulation test. All but one patient evaluated by one test only in the present study had performed the spontaneous nocturnal test. Children evaluated by one test only tended to be younger than the other children and they had lower pubertal stages (see **Table 1**). Reluctance to the IV injection of insulin might be a reason for choosing to evaluate a child with the nocturnal test only.

We also found a highly variable frequency (6–42%) of divergent results from AITTs and nocturnal spontaneous GH tests, which was significantly associated with cut-off values applied (see **Figure 2**). This association is not surprising, since fewer patients will fail lower cut-offs and vice versa, but—as far as we know—this association has never been shown before in children. Further, we showed for the first time lower frequencies of divergent results than 14%. But two previously reported frequencies of divergent results were outside of the range of our results (53% of the children (9) and at least 46% (14). All cut-off levels studied here have been suggested previously in the literature or have been used in clinical practice (8, 9, 16, 17, 21). Besides using previously suggested cut-offs, we calculated the best cut-offs for the nocturnal spontaneous test based on our study population and different cut-offs applied in the AITT. All calculated cut-offs for peak GH levels in the spontaneous test were higher than the corresponding cut-offs in the AITT (see **Table 3**). However, even when using these calculated cut-offs in the spontaneous test together with their corresponding cut-offs in the AITT, the frequencies of divergent results were still high (9–30%), suggesting that these two tests are not interchangeable but rather complimentary.

Another possible explanation for divergent results than cut-off values applied is refractoriness during the stimulation test (15, 22, 23, 28). However, the literature on refractoriness for GH secretion in humans is strikingly sparse. To the best of our knowledge, we found for the first time in the present study evidence for a refractory period in short children. A significantly higher proportion of children with a late spontaneous GH peak failed the AITT. According to our results, the refractory period seems to have a duration of approximately 2 h. In addition, it seems like the time-point for the latest spontaneous GH peak is more important for the difficulties in passing the AITT than the magnitude of it, since the magnitude of the latest spontaneous GH peak was higher in children passing the AITT than in children failing the AITT. In order to not over diagnose GHD in children, refractoriness during the stimulation test needs to be considered.

Still another possible explanation for divergent results in the two GH tests might be that all concentrations above a certain level (3 or 5 µg/L perhaps) only reflect normal fluctuations in healthy and short children. This hypothesis needs to be verified in future studies but is supported by existing normative data (8, 20, 29, 30), by the finding of similar responses to rhGH therapy in short children with stimulated peak values of 5–10 µg/L as in children with stimulated peak values > 10 µg/L (31), and by our finding of similar clinical characteristics in children with and without GH peaks > 7 µg/L except for weight and body mass index (BMI) SDS (see **Table 2**). Children passing both GH tests (Group A) had lower weight SDS, which is in accordance with previous findings showing higher GH levels in thinner children (32, 33).

According to our findings on divergent results and assuming that only one high GH value is enough for GH sufficiency, 7% of short children would be diagnosed with GHD if 7 µg/L was applied as the cut-off and the child was evaluated with the nocturnal test only, even though an AITT would find peaks > 7 µg/L. Furthermore, 19% of short children would be diagnosed with GHD if the same cut-off level was applied and only the AITT was performed, even though a nocturnal test would find peaks > 7 µg/L. The diagnosis of GHD in childhood is multifaceted and can be established without GH testing in some circumstances (3), but in many cases, GH testing is needed, and performing a nocturnal spontaneous test in addition to a stimulation test is probably a valuable supplement. The present study shows that it is possible to perform both tests in a clinical setting.

Possible limitations of the present study were the lack of information on final height and on the effects of rhGH therapy, which decreased our ability to state that children with divergent results did not have GHD and would not benefit from rhGH treatment. However, neurosecretory dysfunction, i.e., poor nocturnal spontaneous secretion but normal stimulated GH secretion (34), is debated and controversial and may not be a common pathological condition (3, 16). Furthermore, normative data on both stimulated and nocturnal spontaneous GH peaks in children include low values (8, 20, 30). In addition, the effect of rhGH therapy on height in idiopathic short stature (ISS) is already known (3). Even though a large proportion of the children in the present study were diagnosed with GHD in the clinical setting according to the responsible pediatric endocrinologist, it is probable that most of them did not have “true” or severe GHD using more stringent criteria. The GHD diagnoses reported on here should be interpreted in view of the fact that ISS is not an approved indication for rhGH therapy in Europe, national and international guidelines valid during the study time period including periods when high cut-off levels were used, and the well-known difficulties in reaching a correct GHD diagnosis. The strengths of this study include the study design; all children were evaluated with both tests in the same manner, i.e., the nocturnal test was immediately followed by the stimulation test the same morning. This eliminated the risk that changes in BMI, pubertal stage, or GH assay could have influenced our results. Other strengths are the population-based setting, decreasing patient selection bias, and that the study reflects the common real-life experience of GH evaluations.

In summary, we evaluated GH testing over 24 years at our center and found that children diagnosed with GHD more often had been evaluated with one GH test only. When the nocturnal spontaneous GH test and the AITT were performed in sequence, the frequency of divergent results was highly variable and dependent on the selected cut-off level. We also found evidence of refractoriness in short children, which may partly explain divergence of the two GH tests. Taken together, these findings suggest that the risk of GHD over diagnosis can be decreased if a nocturnal spontaneous test is added to the GH stimulation test during the evaluation of children with short stature.

## Data Availability Statement

The raw data supporting the conclusions of this article will be made available by the authors, without undue reservation.

## Ethics Statement

The studies involving human participants were reviewed and approved by The Regional Ethics Board at Uppsala, Sweden. Written informed consent from the participants’ legal guardian/next of kin was not required to participate in this study in accordance with the national legislation and the institutional requirements.

## Author Contributions

OL retrieved all data from the medical records, performed the statistical analyses, generated the figures, and wrote the manuscript. ON participated in the study design, interpretation of the findings, and writing of the manuscript. ML was responsible for the study design and the interpretation of the findings, performed some statistical analyses, and finalized the manuscript. All authors contributed to the article and approved the submitted version.

## Funding

The authors declare that this study received funding from ALF funding from the Region Örebro County, the Swedish Research Council (project K2015–54X-22 736–01–4 & 2015-02227); the Swedish Governmental Agency for Innovation Systems (Vinnova) (2014-01438); the Marianne and Marcus Wallenberg Foundation; Inga Britt and Arne Lundberg’s Research Foundation; the Builder Olle Engkvist Foundation; the Swedish Society of Medicine; the Erik and Edith Fernström Foundation for Medical Research; The Foundation for Medical Research at Örebro University Hospital (Nyckelfonden); the Foundation Frimurare Barnhuset in Stockholm; the Stockholm County Council; Karolinska Institutet, Stockholm, Sweden; and Örebro University, Örebro, Sweden. No funder had any role in the design of this study, data collection, data analysis, decision to publish nor in the preparation of the manuscript.

## Conflict of Interest

ON has received speaker´s honoraria from Pfizer, Lilly, Abbott, and Biomarin; consulting fees from Ascendis and Kyowa Kirin; and research support from Kyowa Kirin and the Novo Nordisk Foundation.

The remaining authors declare that the research was conducted in the absence of any commercial or financial relationships that could be construed as a potential conflict of interest.
