# Supplementary material for: Discordance Between Stimulated and Spontaneous Growth Hormone Levels in Short Children Is Dependent on Cut-Off Level and Partly Explained by Refractoriness
Source: Front Endocrinol (Lausanne). 2020 Nov 17;11:584906. doi: 10.3389/fendo.2020.584906 (PMC7705110; doi:10.3389/fendo.2020.584906)
Supplement: Supplementary file 1 [file Table_1.docx]

**Supplementary material**

**Table S1*.* Codes used for the identification of patients in the hospital registries.**

| **Codes for diagnoses** | | |
| --- | --- | --- |
| **Years** | **List used** | **Codes** |
| 1993-1997 | ICD-9 | 253C Panhypopituitarism  253D Pituitary short stature, growth hormone deficiency  253E Other disorders of the pituitary frontal lobe  253H Iatrogenic dysfunction of the pituitary  253X Unspecified disturbances of the pituitary  253W Other disorders of the pituitary  259E Short stature which is not classified  783E Absence of expected normal physiological development, failure to thrive, short stature growth |
| 1997- | ICD-10 | E34.3 Short stature  E23.0 Growth hormone deficiency, hypofunction of the pituitary |
| **Codes for procedures** | | |
| **Years** | **List used** | **Codes** |
| 1993-1997 | “The 9000-list” | 9205 |
| 1997-2006 | “TÅL” | V9205 |
| 2007- | “KVÅ” | AB007 Arginine test  AB012 GHRH test  AB0013 Glucagon test  AB017 Insulin test  AB018 Clonidine test  AB019 L-dopa test  AB034 Hormone profile, GH sampling  AB035 Hormone profile, GH 24-hour sampling with pump  AB036 Hormone profile, GH 24-hour sampling without pump  AB052 Other specified endocrine tests  AB053 Other specified hormone samplings |

ICD, International statistical classification of diseases and related health problems. “The 9000-list”, “TÅL” and “KVÅ” are lists of classifications of medical procedures used at the hospital during different periods.

**Table S2***.* **Assay methods and standard preparations used for the analysis of growth hormone concentrations during the study period.**

| Time period | Assay | Standard preparation |
| --- | --- | --- |
| 1^st^ of March 1991 – 20^th^ of April 1993 | hGH RIA method (Pharmacia®) | IRP 80/505 |
| 20^th^ of April 1993 – 14^th^ of February 2000 | Delfia (Wallac®) | IRP 80/505 |
| 15^th^ February 2000 – 1^st^ of March 2009 | AutoDelfia (Wallac®) | IRP 80/505 |
| 2^nd^ of March 2009 – 18^th^ of January 2010 | Immunlite (Siemens®) | IRP 98/574 |
| 18^th^ of January 2010 – | Immunlite1000 (Siemens®) | IRP 98/574 |

hGH, human growth hormone. IRP, international reference preparation. RIA, radio immunoassay.

**Data capture form**

Code: _________

**Background information**

A copy of the child's growth curve, marked with the child's code, is collected:

Yes No

Sex: Girl Boy

Mother's height: _________________________

Father's height: __________________________

Ethnicity (or language used at the visits): __________________________________________

Medical history and prior medical therapy: _______________________________________

______________________________________________________________________________________________________________________________________________________

Has the child been exposed to cranial irradiation? Yes No Not known

Tanner pubertal stage including testicular volume: __________________________________

___________________________________________________________________________

Date for the assessment of pubertal stage: ________________________________________

Reason(s) for the GH evaluation: ________________________________________________

___________________________________________________________________________

Name of the physician responsible for the medical evaluation of the patient: ___________________________________________________________________________

**GH evaluation**

Did the patient receive sex steroid priming prior to GH testing?

Yes No Not known

If so, what type and dose? _____________________________________________________

Date for the GH evaluation: __________________________________

Age at the GH evaluation: _­­­­­­__________ years ___________ months.

Height at the GH evaluation: ____________________________________________________

Weight at the GH evaluation: ___________________________________________________

Sitting height at the GH evaluation: ______________________________________________

Nocturnal spontaneous GH secretion

How many blood samples were sent to the laboratory for the analysis of nocturnal spontaneous GH secretion? ___________________________________________________

Number of samples with hemolysis during the nocturnal spontaneous test: _____________

Calculated mean value of all GH concentrations (8 PM–8 AM. Do not include uncertain values due to, for example, hemolysis): ___________________________________________

Number of peaks during the night? ____________________________

Specify the GH concentrations at these peaks (all peaks, not only the maximum peak):
___________________________________________________________________________

Specify the lowest GH concentration during the night? ______________________________

Which unit was used for GH concentrations? mIU/L µg/L

Stimulated GH secretion

Which stimuli were given and what doses were used? _______________________________

___________________________________________________________________________

Enter the highest GH concentration after stimulation: _______________________________

Did the highest GH concentration occur after the first or the second stimulus? ___________

Adverse events to stimulus given? _______________________________________________

If insulin was given What was the lowest p-glucose value? _________________

What was the highest S-cortisol value? _________________

**Assessment of the GH evaluation and follow-up**

After GH testing, was the patient diagnosed with GH deficiency?

Yes No Unclear

Was the patient offered rhGH treatment? Yes No Unclear

How many doctors were involved in the decision on offering rhGH therapy?

One doctor Two doctors > Two doctors Unclear

If the patient was offered rhGH therapy, but not diagnosed with GH deficiency, what was the reason for rhGH therapy? (e.g., SGA without catch up, Turner syndrome) ________________

___________________________________________________________________________

If the patient was diagnosed with GH deficiency, what was the etiology for the deficiency? ___________________________________________________________________________

___________________________________________________________________________

Which further investigations were performed in patients diagnosed with GH deficiency? (e.g., MRI, genetic testing and/or evaluation of other pituitary hormones) ______________

___________________________________________________________________________

___________________________________________________________________________

Was the patient followed up at the Department of Pediatrics, Örebro University Hospital, after GH testing?

Yes No Unclear

If so, for what reason? _________________________________________________________

Enter other valuable information from the medical records (e.g., rhGH therapy was given for X number of years, or the patient was admitted to another medical service for continued follow-up of growth velocity but was re-admitted because of poor growth a few years later).

____________________________________________________________________________________________________________________________________________________________________________________________________________________________________________________________________________________________________________

Enter information from the last visit at the Department of Pediatrics, Örebro University Hospital.

Date: ____________ Age: _____________ Height: _____________ Weight: _____________

Sitting height: _____________ Pubertal stage: _____________

**List of additional variables used for data collection from the medical records**

- GH concentration at the time of the last spontaneous peak.
- All time points for the spontaneous GH peaks.
- GH concentration at the time of the last peak during AITT.
- All time points for GH peaks during AITT.
- IGF-1 concentration prior to GH testing (#1).
- Date for IGF-1 testing prior to GH testing (#1).
- Unit for IGF-1 concentration prior to GH testing (#1).
- Reference interval for IGF-1 concentration prior to GH testing (#1).
- IGF-1 value prior to GH testing interpreted as low, normal or high (#1).
- Which laboratory performed the analysis of the IGF-1 value prior to GH testing? (#1).
- IGF-1 concentration prior to GH testing (#2).
- Date for IGF-1 testing prior to GH testing (#2).
- Unit for IGF-1 concentration prior to GH testing (#2).
- Reference interval for IGF-1 concentration prior to GH testing (#2).
- IGF-1 value prior to GH testing interpreted as low, normal or high (#2).
- Which laboratory performed the analysis of the IGF-1 value prior to GH testing? (#2)
- IGF-1 concentration at the time of GH testing.
- Date for IGF-1 testing at the time of the GH testing.
- Unit for IGF-1 concentration at the time of the GH testing.
- Reference interval for IGF-1 concentration at the time of the GH testing.
- IGF-1 value at the time of GH testing interpreted as low, normal or high.
- Which laboratory performed the analysis of the IGF-1 value at the time of the GH testing?
- IGF-1 concentration after GH testing (#1).
- Date for IGF-1 testing after GH testing (#1).
- Unit for IGF-1 concentration after the GH testing (#1).
- Reference interval for IGF-1 concentration after GH testing (#1).
- IGF-1 value after GH testing interpreted as low, normal or high (#1).
- Which laboratory performed the analysis of the IGF-1 value after the GH testing? (#1)
- IGF-1 concentration after GH testing (#2).
- Date for IGF-1 testing after GH testing (#2).
- Unit for IGF-1 concentration after the GH testing (#2).
- Reference interval for IGF-1 concentration after GH testing (#2).
- IGF-1 value after GH testing interpreted as low, normal or high (#2).
- Which laboratory performed the analysis of the IGF-1 value after the GH testing? (#2)
- IGF-1 concentration after GH testing (#3).
- Date for IGF-1 testing after GH testing (#3).
- Unit for IGF-1 concentration after the GH testing (#3).
- Reference interval for IGF-1 concentration after GH testing (#3).
- IGF-1 value after GH testing interpreted as low, normal or high (#3).
- Which laboratory performed the analysis of the IGF-1 value after the GH testing? (#3)
- Last IGF-1 concentration after GH testing noted in the patient’s medical record.
- Date for the last IGF-1 concentration after GH testing.
- Unit for the last IGF-1 concentration after GH testing.
- Reference interval for the last IGF-1 concentration after GH testing.
- The last IGF-1 value interpreted as low, normal or high.
- Which laboratory performed the last IGF-1 analysis?
- Date for evaluation of bone age.
- Was the bone age evaluation performed by a radiologist or by computer automation?
- The name of the radiologist evaluating the bone age.
- What was the bone age according to Greulich-Pyle evaluated by a radiologist?
- What was the bone age according to Greulich-Pyle evaluated by BoneXpert?
- Chronological age at the time of bone age evaluation.
- Bone age standard deviation scores.
- Which percentage of predicted final height had the child already achieved at the time of bone age evaluation?
- What was the final height prognosis based on bone age?
- Date for start of rhGH therapy.
- Name of rhGH prescribed.
- Initial dose of rhGH.
- Changes in the rhGH dose during the treatment period, including final dose.
- Date for termination of rhGH therapy.
- Was rhGH therapy stopped once final height was achieved?
- Which rhGH dose was used at the time of discontinuation of therapy?
- Was GH re-testing performed after the completion of rhGH therapy?
- Was the patient referred to the endocrine department for adults for follow-up?
- Height at the end of the rhGH therapy.
- Age at the end of the rhGH therapy.
- Pubertal stage at the end of rhGH therapy.
